# Supplementary material for: Comparative multiomics analysis of cell physiological state after culture in a basket bioreactor
Source: Sci Rep. 2022 Nov 23;12:20161. doi: 10.1038/s41598-022-24687-4 (PMC9686226; doi:10.1038/s41598-022-24687-4)
Supplement: Supplementary file 1 — Supplementary Information 1. [file 41598_2022_24687_MOESM1_ESM.zip › raw data/Transcriptomic raw data/7.AS/2.dasplot/TE1vsTC1/RI/15_ANP32B_NW_023666038.1_38450340_38450539_-@NW_023666038.1_38447839_38450539_-@NW_023666038.1_38447839_38449714_-.pdf]

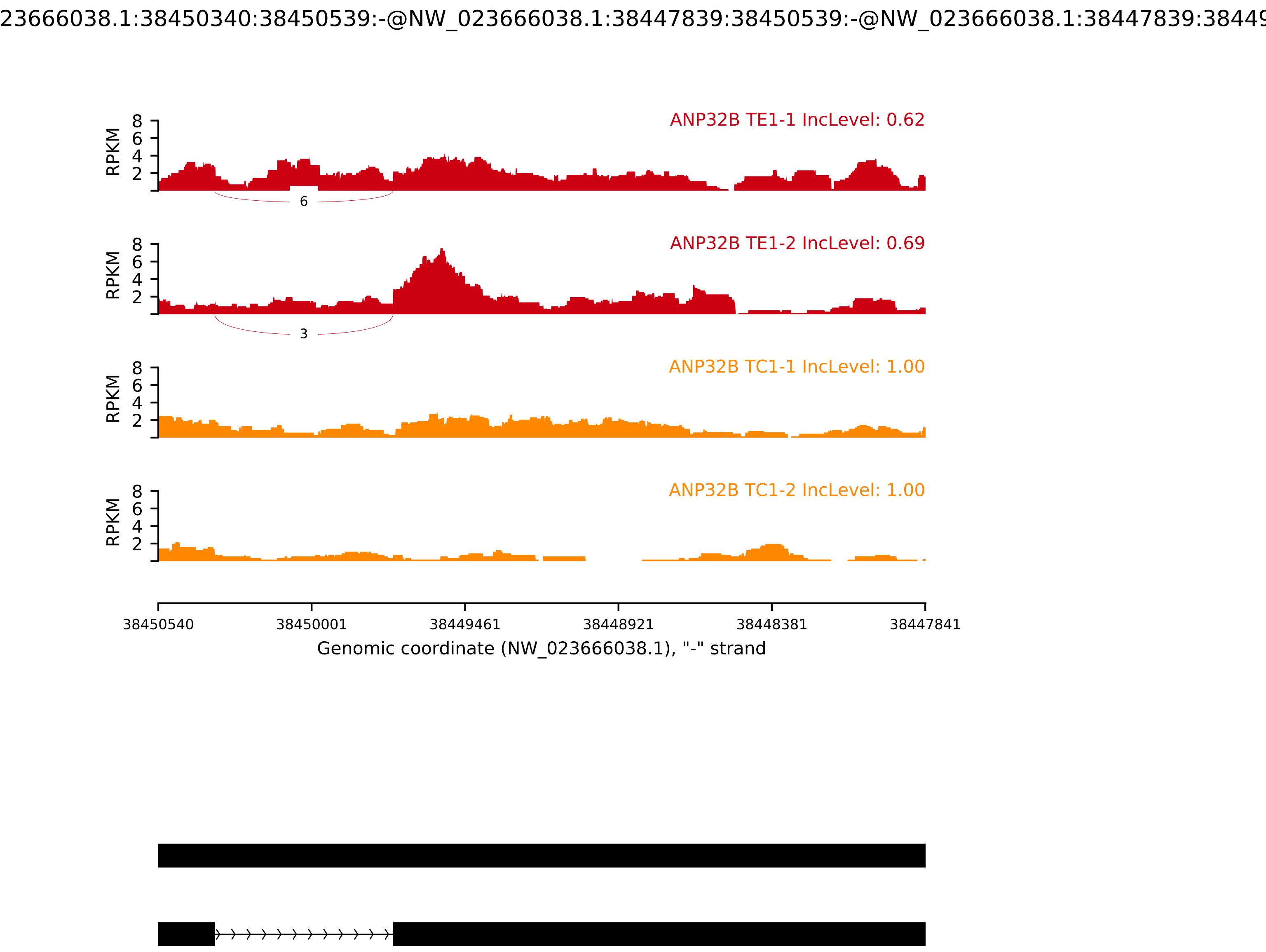
23666038.1:38450340:38450539:-@NW_023666038.1:38447839:38450539:-@NW_023666038.1:38447839:3844[illegible]
ANP32B TE1-1 IncLevel: 0.62
RPKM
8
6
4
2
6
ANP32B TE1-2 IncLevel: 0.69
RPKM
8
6
4
2
3
ANP32B TC1-1 IncLevel: 1.00
RPKM
8
6
4
2
ANP32B TC1-2 IncLevel: 1.00
RPKM
8
6
4
2
38450540
38450001
38449461
38448921
38448381
38447841
Genomic coordinate (NW_023666038.1), "-" strand
